# Supplementary material for: Silicon Nanowire Field-Effect Transistor as Label-Free Detection of Hepatitis B Virus Proteins with Opposite Net Charges
Source: Biosensors (Basel). 2021 Nov 10;11(11):442. doi: 10.3390/bios11110442 (PMC8615946; doi:10.3390/bios11110442)
Supplement: Supplementary file 1 [file biosensors-11-00442-s001.zip › biosensors-1405191-supplementary.pdf]

biosensors

Supplementary Materials

Silicon Nanowire Field-Effect Transistor as Label-Free Detection of Hepatitis B Virus Proteins with Opposite Net Charges

Suh Kuan Yong [1], Shang-Kai Shen [2], Chia-Wei Chiang [2], Ying-Ya Weng [2], Ming-Pei Lu [3,*] and Yuh-Shyong Yang [1,*]

[1] Department of Biological Science and Technology, National Yang Ming Chiao Tung University, 300 Hsinchu, Taiwan; wendyyongsk.bt05g@nctu.edu.tw
[2] Institute of Biomedical Engineering, National Yang Ming Chiao Tung University, 300 Hsinchu, Taiwan; fr509.cm05g@nctu.edu.tw (S.-K.S.); chiangwill0813@gmail.com (C.-W.C.); lynneweng8571@gmail.com (Y.-Y.W.)
[3] Taiwan Semiconductor Research Institute, National Applied Research Laboratories, 300 Hsinchu, Taiwan
* Correspondence: mplu@narlabs.org.tw (M.-P.L.); ysyang@mail.nctu.edu.tw (Y.-S.Y.)

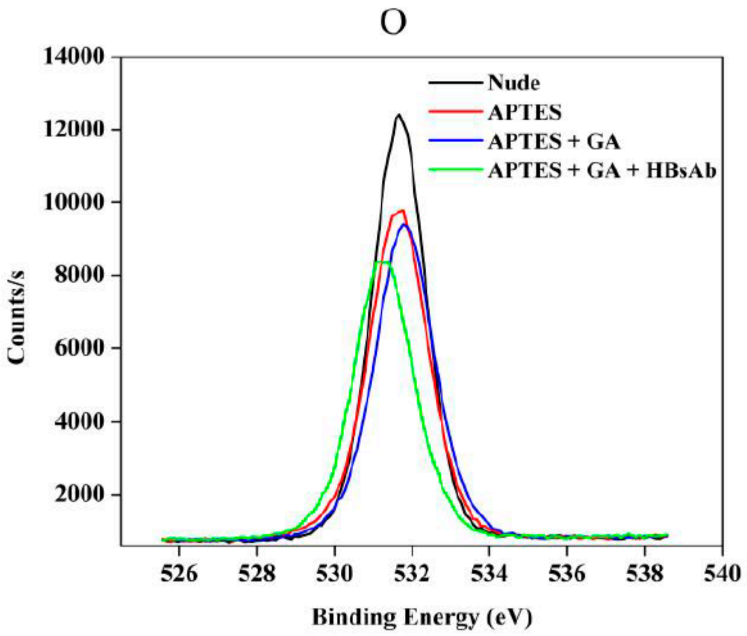

Figure S1. XPS oxygen spectra of four silicon wafer (Si-wafer) surface modifications. Nude indicates the Si-wafer without surface modification, APTES indicates after treated with 2% APTES, APTES + GA indicates after treated with 2% APTES and 2.5% glutaraldehyde, and APTES + GA + HBsAb indicates after treated with 2% APTES and 2.5% glutaraldehyde and one μg/mL of HBsAb.

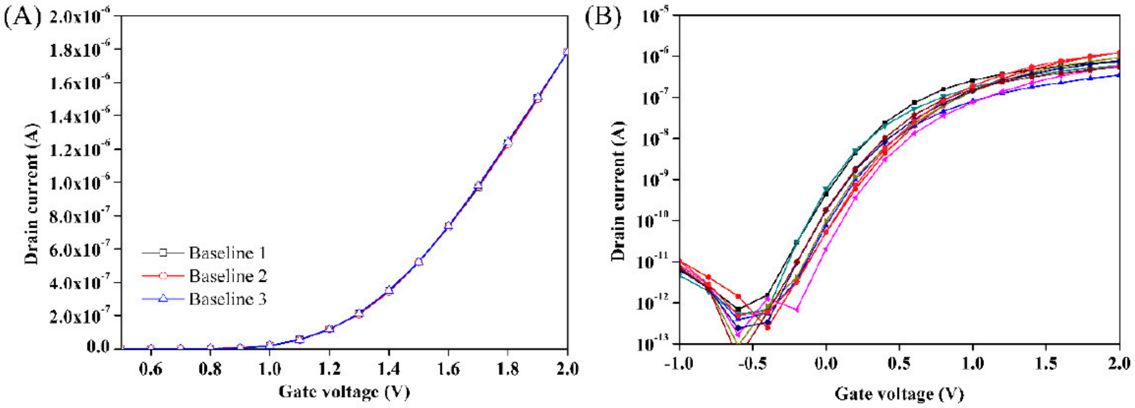

Figure S2. The electrical properties of three baselines of one functionalized device and nine nude devices. (**A**) The baseline electrical property of a functionalized device was conducted three times at a fixed drain voltage (VD = 0.5 V), and gate voltage sweeps from 0.6 to 2.0 V. (**B**) The electrical property of the nine devices were conducted at a fixed drain voltage (VD = 0.5 V), and gate voltage sweeps from −1 to 2.0 V. The data was plotted in ID-VG curve.

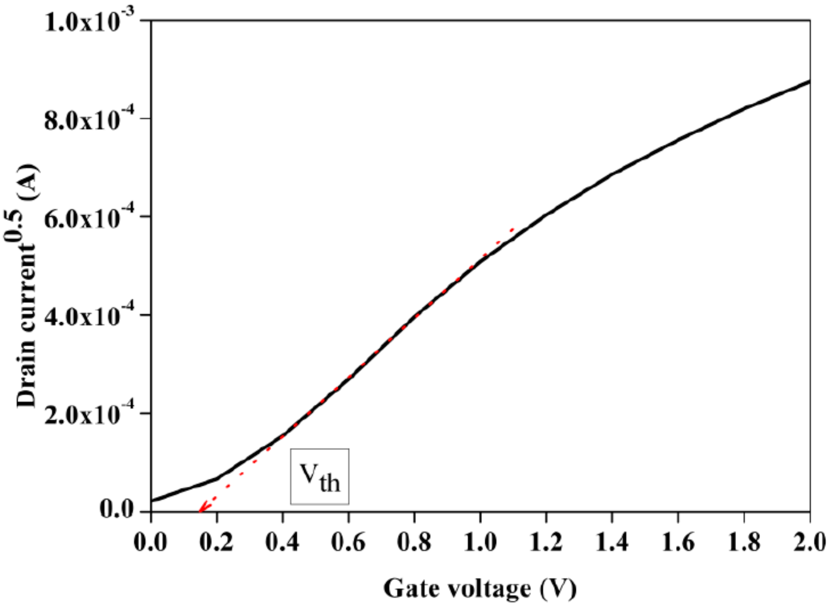

Figure S3. The extraction of threshold voltage of pSiNWFET. The Vth is extracted at the interception of the linear approximation of ID-VG curve at the maximum slope point.

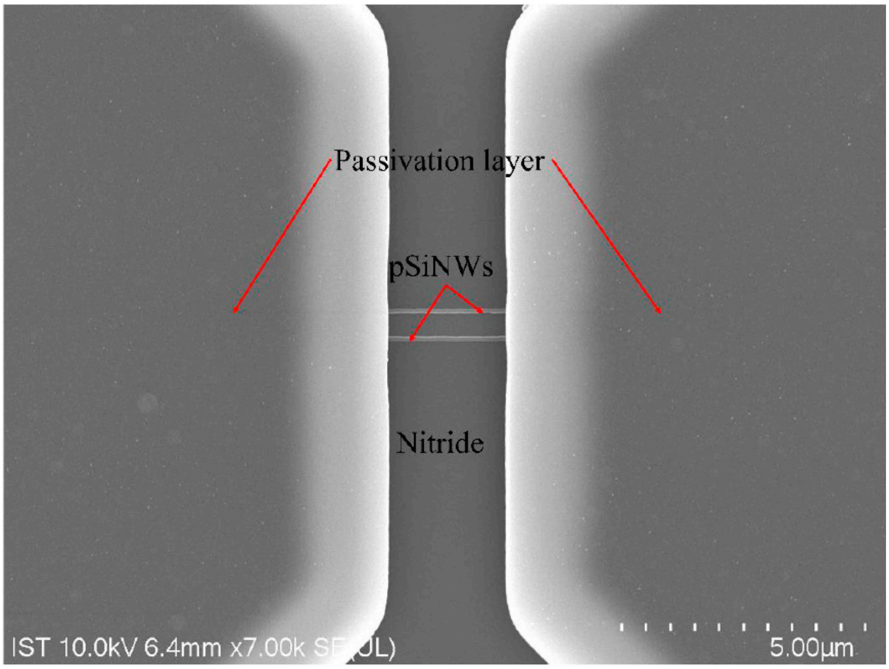

Figure S4. The top-view SEM image of the device. There are two NWs located on the nitride surface and in between passivation layer.

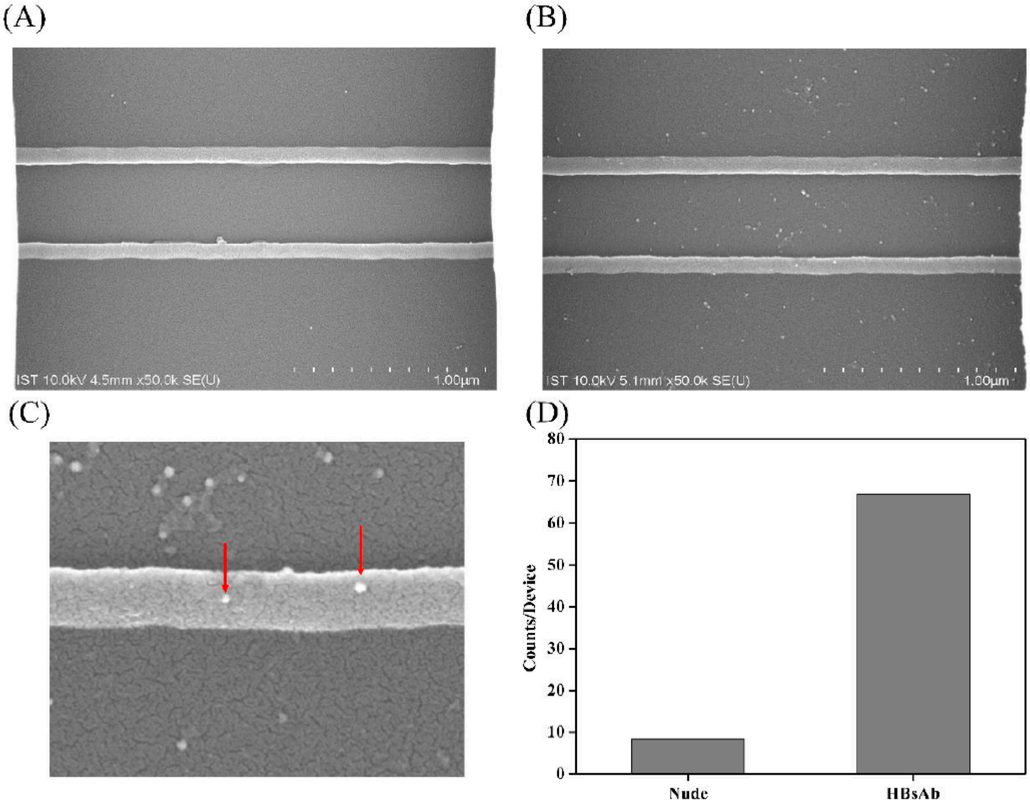

Figure S5. The SEM images of the HBsAb immobilization process. The pSiNW was treated with and without a series of the surface modification process and observed using SEM after the addition of nanogold-conjugated IgG. (**A**) The SEM image of two pSiNWs treated without surface modification treatment (Nude). (**B**) The SEM image of two pSiNWs treated with surface modification and HBsAb immobilization (HBsAb). The nanogold particles were observed and analyzed using ImageJ software. (**C**) In the enlarged image of Figure 2SB, red arrows were pointing at some of the nanogold particles. (**D**) All devices were analyzed using ImageJ software, and the counts/device of each set was plotted, the error bar indicates standard error from three devices.
